# Predicting permanent hypoparathyroidism after thyroidectomy: role of early postoperative parathyroid hormone levels and surgical factors

**DOI:** 10.1186/s12902-026-02369-w

**Published:** 2026-06-25

**Authors:** Ahmet Akmercan, Kerim Deniz Batun, Ahmet Hakan Atalay, Muhammer Ergenç, Bahadir M. Güllüoğlu, M. Ümit Uğurlu

**Affiliations:** 1 Department of General Surgery, Marmara University Pendik Training and Research Hospital, Istanbul, Turkey; 2 Department of General Surgery, Marmara University School of Medicine, Istanbul, Turkey; 3 Breast and Endocrine Surgery Unit, Department of General Surgery, Marmara University School of Medicine, Istanbul, Turkey; 4 Marmara University School of Medicine, Başıbüyük Campus Başıbüyük Mah, Maltepe Başıbüyük Yolu Sok. No: 9 / 2 Maltepe, Istanbul, 34854 Turkey

**Keywords:** Hypocalcemia, Hypoparathyroidism, Incidence, Risk factors, Thyroidectomy

## Abstract

**Background:**

This study aimed to determine the incidence of permanent hypoparathyroidism (PH) after thyroidectomy and evaluate the association between PH and perioperative surgical, clinical, and biochemical factors.

**Methods:**

Patients who underwent total or completion thyroidectomy between 2017 and 2024 were retrospectively evaluated. PH was defined as a serum parathyroid hormone (PTH) level < 15 pg/mL and the need for active vitamin D, with or without calcium supplementation, to prevent hypocalcemic symptoms persisting for at least 6 months postoperatively. Patients were categorized according to PH status, and clinical, surgical, and biochemical parameters were analyzed.

**Results:**

Among the 498 eligible patients, 18 (3.6%) developed PH. A postoperative day 1 PTH level ≤ 7 pg/mL predicted PH with 86.7% sensitivity and 90.3% specificity and was an independent risk factor in multivariate analysis (odds ratio [OR]: 16.60, *p* < 0.001). Completion thyroidectomy was also independently associated with PH (OR: 6.14, *p* = 0.027). Postoperative day 1 calcium ≤ 8.3 mg/dL (OR: 14.20, *p* < 0.001) and the presence of parathyroid tissue in the surgical specimen (OR: 6.25, *p* < 0.001) were significant in univariate analysis but not in multivariate analysis (*p* = 0.08 and *p* = 0.056, respectively).

**Conclusions:**

Permanent hypoparathyroidism remains a clinically significant complication of thyroidectomy, with an incidence of 3.6% in our cohort. A postoperative day 1 PTH ≤ 7 pg/mL was independently associated with PH and demonstrated an excellent negative predictive value (99.4%), supporting its clinical utility for ruling out PH rather than confidently predicting it. Completion thyroidectomy was also independently associated with PH. Given the limited number of events, these findings warrant validation in larger, prospective cohorts.

**Clinical trial number:**

Not applicable.

## Introduction

Hypoparathyroidism is a common complication following thyroidectomy. It is typically attributed to unintentional harm to the parathyroid glands and/or their blood vessels [1, 2]. Although most patients with postoperative hypoparathyroidism have only transient hypoparathyroidism, a notable and varied percentage of patients are diagnosed with permanent hypoparathyroidism (PH). High-volume, single-center studies reported an incidence of under 5% for PH, while subsequent nationwide multicenter studies found an incidence rate as high as 16.7% [3–6].

Parathyroid hormone (PTH) and serum calcium levels are the main regulators of calcium homeostasis and directly reflect parathyroid gland function. Both parameters are routinely measured in the early postoperative period following thyroid surgery and are widely used to assess parathyroid recovery and stratify the risk of hypoparathyroidism. Several studies have demonstrated that early postoperative serum PTH levels are predictive of both transient and permanent hypocalcemia and have emphasized that PTH should be interpreted in combination with serum calcium levels for optimal management of postoperative hypocalcemia [7–9].

This study aimed to determine the incidence of PH and assess the association between surgical, medical, and postoperative biochemical factors, particularly PTH and serum calcium, and the development of PH after thyroidectomy.

## Materials and methods

### Study design, patient characteristics, and definitions

Patients aged ≥ 18 years who underwent total or completion thyroidectomy for any reason between 2017 and 2024 were enrolled in this retrospective study. Ethical approval was obtained from the Medical Ethics Committee of Marmara University (approval number: 09.2024.1071), and the study was reported following the STROCSS guidelines [10].

Patients were excluded if they had a documented preoperative parathyroid disorder, were using active vitamin D or calcium for any reason, or lacked six-month postoperative follow-up data. We assessed the operative and postoperative outcomes of patients who underwent thyroidectomy. Demographics (age, sex, body mass index [BMI]), surgical indications, details regarding the extent of surgery, length of hospital stay, histopathological data (thyroid size and presence of parathyroid glands in the surgical specimen), and information on adjuvant radioiodine therapy were collected.

Postoperative first-day and sixth-month serum parathyroid hormone (PTH) and calcium levels were retrieved from an electronic database. Data regarding the use of calcium and/or active vitamin D treatment at six months postoperatively were obtained from the patients’ medical records. Serum calcium (Beckman Coulter analyzer, reference range: 8.6–10 mg/dL) and serum PTH (Beckman Coulter analyzer, reference range: 15–65 pg/mL) levels were measured using a standardized immunoassay. PH was defined as a serum PTH level < 15 pg/mL and the requirement for active vitamin D, with or without calcium supplementation, to prevent hypocalcemia symptoms for at least 6 months after surgery [11–13].

### Surgical procedure

All thyroid surgeries were performed by high-volume endocrine surgeons. A surgeon performing > 50 thyroidectomies annually was considered a high-volume endocrine surgeon. At our center, > 100 thyroidectomies are performed annually, which is considered a high-volume center [14].

All thyroidectomies were performed using a standardized approach. Following superior vessel ligation and upper pole mobilization, a capsular dissection technique was used to preserve the parathyroid glands. The recurrent laryngeal nerve was identified, and the inferior vessels of the thyroid gland were ligated. The vascular structures were ligated using a standard knot-tying method. Energy devices were used for dissection. Central lymph node dissection (CLND) or lateral lymph node dissection (LLND) was performed when indicated. The surgical site and thyroidectomy specimens were routinely examined after thyroidectomy. In patients in whom the parathyroid glands were accidentally resected, they were auto-transplanted into either the sternocleidomastoid or strap muscles.

### Statistical analyses

Descriptive statistics were used to summarize the demographic and clinicopathological characteristics of the study cohort. Categorical variables are presented as frequencies and percentages [n (%)], whereas continuous variables are expressed as median and interquartile range (IQR). The distribution of continuous variables was assessed using the Shapiro–Wilk test.

Categorical variables were compared using the chi-square test or Fisher’s exact test, as appropriate, whereas differences between groups for non-normally distributed continuous variables were analyzed using the Mann–Whitney U test. Receiver operating characteristic (ROC) curve analysis was performed to determine the optimal cutoff values for the first postoperative day serum parathyroid hormone and calcium levels in predicting permanent hypoparathyroidism. The sensitivity, specificity, and positive and negative predictive values (PPV and NPV) were calculated for these thresholds.

Multivariate logistic regression analysis was conducted to identify the independent predictors of permanent hypoparathyroidism. Odds ratios (ORs) with corresponding 95% confidence intervals (CIs) were reported for the results. Statistical significance was set at *p* < 0.05. All statistical analyses were performed using the Jamovi software (version 2.5.3).

## Results

A total of 525 patients underwent total or completion thyroidectomy between January 2017 and June 2024 at our institution. Finally, 498 patients were eligible for enrollment in the study. The baseline characteristics and clinicopathological features of the patients are summarized in Table 1. The median age was 50 (IQR 39–58), and most of the patients were female (*n* = 376, 75.5%). The most frequent indication for surgery was thyroid carcinoma (*n* = 243, 48.8%). Of the patients, 458 (92%) underwent total thyroidectomy, and 40 (8%) underwent completion thyroidectomy. In total, 18 of 498 (3.6%) patients were diagnosed with PH. No significant differences were found between patients who developed PH and those who did not in terms of sex, age, BMI, duration of surgery, CLND, thyroid size, and preoperative diagnosis. PH developed in 14 (3.1%) of 458 patients who underwent total thyroidectomy and in 4 (10.0%) of 40 patients who underwent completion thyroidectomy (*p* = 0.024). Patients with PH showed significantly higher rates of parathyroid gland presence in the surgical specimens (55.6% vs. 17.9%, *p* < 0.001).

**Table 1:** Comparison of clinicopathological characteristics between patients with and without permanent hypoparathyroidism

Variables	All patients (n=498)	Hypoparathyroidism negative (n=480)	Hypoparathyroidism positive (n=18)	*p*-value
Sex, n (%)				
Male	122 (24.5%)	118 (24.6%)	4 (22.2%)	0.819
Female	376 (75.5%)	362 (75.4%)	14 (77.8%)	
Age (years), median (IQR)	50 (39-58)	50 (39-58)	46 (41-53)	0.461
BMI, kg/m^2^, median (IQR)	27.7 (24.5-32)	27.8 (24.6-32)	24.7 (23.2-30.5)	0.309
Preoperative diagnosis, n (%)				
Multinodular goiter	120 (24.1%)	115 (24.0%)	5 (27.8%)	0.664
Graves' disease	44 (8.8%)	43 (9.0%)	1 (5.6%)	
Carcinoma	243 (48.8%)	236 (49.2%)	7 (38.9%)	
Other	91 (18.3%)	86 (17.9%)	5 (27.8%)	
Surgery type, n (%)				
Total thyroidectomy	458 (92.0%)	444 (92.5%)	14 (77.8%)	**0.024**
Completion thyroidectomy	40 (8.0%)	36 (7.5%)	4 (22.2%)	
Duration of surgery (min), median (IQR)	104 (90-120)	100 (90-120)	120 (100-134)	0.191
Central lymph node dissection, n (%)				
Yes	52 (10.4%)	49 (10.2%)	3 (16.7%)	
No	446 (89.6%)	431 (89.8%)	15 (83.3%)	0.822
Thyroid size, mm, median (IQR)	60 (50-80)	60 (50-80)	60 (40-78.8)	0.65
Presence of parathyroid glands in the specimen, n (%)				
Yes	96 (19.3%)	86 (17.9%)	10 (55.6%)	
No	402 (80.7%)	394 (82.1%)	8 (44.4%)	<**0.001**
First-day postoperative serum calcium level, mg/dL, median (IQR)				<**0.001**
	8.7 (8.3-9)	8.7 (8.3-9.0)	8.1 (7.5-8.4)	
First-day postoperative serum PTH level, pg/mL, median (IQR)	24.4 (14.1-36.9)	25.1 (15.9-37.0)	3.1 (2.6-4.9)	<**0.001**
Length of hospital stay (days), median (IQR)	2 (1-2)	2 (1-2)	2 (2-3)	0.085
Radioiodine ablation, n (%)				
Yes	135 (27.1%)	129 (26.9%)	6 (33.3%)	0.549
No	363 (72.9%)	351 (73.1%)	12 (66.7%)	

Percentages represent column percentages within each outcome group. Permanent hypoparathyroidism rates were 3.1% (14/458) after total thyroidectomy and 10.0% (4/40) after completion thyroidectomy. BMI = body mass index; IQR = interquartile range; PTH = parathyroid hormone

**Fig. 1:**
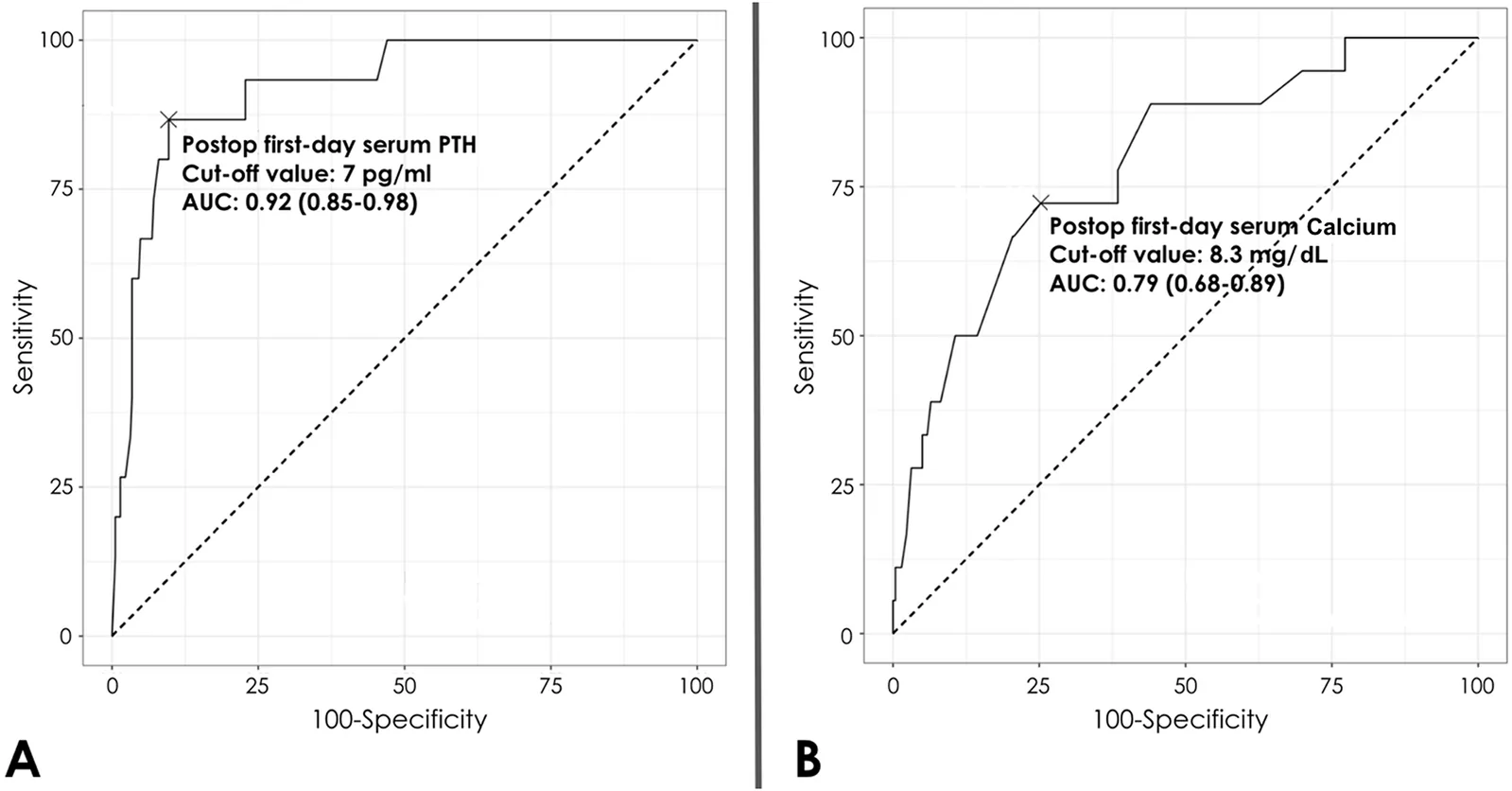
Receiver operating characteristic (ROC) curves for postoperative first-day serum PTH (**A**) and Calcium (**B**) levels as predictors of permanent hypoparathyroidism

The postoperative first day serum calcium (8.1 vs. 8.7 mg/dL, *p* < 0.001) and PTH (3.1 vs. 25.1 pg/mL, *p* < 0.001) levels were significantly lower in patients who developed PH. In the ROC analysis **(**Fig. 1**)**, postoperative first-day PTH levels ≤ 7 pg/mL demonstrated a sensitivity of 86.7% and a specificity of 90.3% for predicting PH (AUC: 0.92, 95% CI: 0.85–0.98, *p* < 0.001). Postoperative first-day calcium levels ≤ 8.3 mg/dL demonstrated a sensitivity of 72.2% and specificity of 74.7% for predicting PH (AUC: 0.79, 95% CI: 0.68–0.89, *p* < 0.001). The sensitivity, specificity, PPV, and NPV for the PTH and calcium level cut-offs on the first postoperative day are shown in Table 2.

**Table 2:** Predictive value of postoperative first day serum PTH and calcium levels for permanent hypoparathyroidism

Variables	Postoperative first day PTH level ≤ 7 pg/mL	Postoperative first day serum calcium level ≤ 8.3 mg/dL
Sensitivity, %	86.7	72.2
Specificity, %	90.3	74.7
Positive predictive value, %	27.7	9.7
Negative predictive value, %	99.4	98.6

PTH = parathyroid hormone

Univariate analysis indicated that lower postoperative serum calcium levels (≤ 8.3 mg/dL) and lower PTH levels (≤ 7 pg/mL) were significantly related to a higher likelihood of PH, with odds ratios (OR) of 14.20 (95% CI: 4.16-50.00, *p* < 0.001) and 33.30 (95% CI: 11.11–100.00, *p* < 0.001), respectively. The presence of parathyroid glands in the specimen (OR: 6.25, 95% CI: 2.12-20.00, *p* < 0.001) and completion of thyroidectomy (OR: 4.55, 95% CI: 1.20-14.37, *p* = 0.014) were also identified as significant predictors of PH.

In the multivariate analysis, low postoperative serum PTH levels (≤ 7 pg/mL) remained independently associated with PH (OR: 16.60, 95% CI: 4.34–100.00, *p* < 0.001). Postoperative serum calcium levels were not found to be a significant factor predicting PH (OR: 3.84, 95% CI: 0.90–5.00, *p* = 0.080). Further, completion thyroidectomy (OR: 6.14, 95% CI: 1.18–34.40, *p* = 0.027) was also independently associated with PH, whereas the presence of parathyroid glands in the specimen (OR: 3.44, 95% CI: 0.97–12.50, *p* = 0.056) did not reach statistical significance (Table 3).

**Table 3:** Univariate and multivariate analyses of factors associated with permanent hypoparathyroidism

Variables	Univariate analyses			Multivariate analyses		
	OR	95% CI	*p*-value	OR	95% CI	*p*-value
First-day postoperative serum calcium level, mg/dL					0.90-5.00	
>8.3	1.00 (ref)		<**0.001**	1.00 (ref)		0.08
≤8.3		4.16-50.00		3.84		
	14.2					
First-day postoperative serum PTH level, pg/mL		11.11-100.00			4.34-100.00	
>7	1.00 (ref)		<**0.001**	1.00 (ref)		<**0.001**
≤7	33.3			16.6		
The presence of parathyroid glands on the specimen		2.12-20.00			0.97-12.50	
No	1.00 (ref)		<**0.001**	1.00 (ref)		0.056
Yes	6.25			3.44		
Surgery type						
Total thyroidectomy	1.00 (ref)	1.20-14.37	**0.014**	1.00 (ref)	1.18-34.40	**0.027**
Completion thyroidectomy	4.55			6.14		

OR = odds ratio; CI = confidence interval; PTH = parathyroid hormone; ref = reference category. Estimates derived from univariate and multivariable logistic regression analyses. Given the limited number of permanent hypoparathyroidism events (n=18), the wide confidence intervals reflect the limited statistical power of the multivariable model and should be interpreted with caution

## Discussion

PH remains one of the most significant and challenging complications of thyroidectomy, with substantial long-term effects on patient outcomes and quality of life, as chronic hypoparathyroidism has been associated with renal, neurological, cardiovascular, and neuropsychiatric complications, as well as increased mortality [15]. In our cohort of 498 patients who underwent total or completion thyroidectomy, we found a 3.6% incidence of PH, consistent with previously noticed ranges in high-volume centers. Our findings demonstrated that early postoperative serum PTH ≤ 7 pg/mL was independently associated with PH with high sensitivity (86.7%) and specificity (90.3%), and showed an exceptionally high negative predictive value (99.4%), supporting its clinical utility for ruling out PH. Furthermore, compared with total thyroidectomy, completion thyroidectomy was also independently associated with permanent hypoparathyroidism.

PH incidence varies widely across published studies, ranging from below 5% in high-volume institutional cohorts [11, 12, 16–19], to up to 28.5% in multicenter and nationwide studies [5, 20–22]. This heterogeneity is primarily attributable to differences in PH definitions — a recent systematic review identified more than 20 distinct definitions of PH and emphasized the need for standardized diagnostic and therapeutic guidelines [23] — as well as in study design and surgical volume. The same cohort can yield substantially different incidence rates depending on the criteria applied, varying with the threshold for serum PTH or calcium and the duration of postoperative supplementation required (six months versus one year) [22, 24]. Using the American Thyroid Association–aligned definition (serum PTH < 15 pg/mL together with sustained need for active vitamin D, with or without calcium, ≥ 6 months postoperatively) [13], we observed a PH incidence of 3.6%, consistent with rates reported by other high-volume single-center cohorts.

Perioperative PTH measurement is widely used for early risk stratification after thyroidectomy and has consistently been shown to predict postoperative hypoparathyroidism across different assay methods and measurement time points [7, 25]. In our cohort, a postoperative first-day PTH threshold of ≤ 7 pg/mL demonstrated high sensitivity (86.7%) and specificity (90.3%) for predicting PH, with a negative predictive value (NPV) of 99.4% and a positive predictive value (PPV) of 27.7%. These findings are consistent with previous studies and recent meta-analyses supporting the prognostic role of early postoperative PTH measurement [8, 26]. The exceptionally high NPV suggests that postoperative first-day PTH measurement may be particularly useful for identifying patients at low risk of developing PH, supporting clinical decision-making by helping clinicians select patients who are unlikely to require prolonged calcium or active vitamin D supplementation and who may be suitable for safer early discharge and less intensive biochemical follow-up. In contrast, although low postoperative first-day PTH levels were significantly associated with PH, the modest PPV (27.7%) limits the ability of a single measurement to confidently predict it; approximately three-quarters of patients with PTH ≤ 7 pg/mL on the first postoperative day will not develop PH. Therefore, postoperative first-day PTH should be interpreted as part of an integrated clinical assessment rather than as an isolated determinant of PH.

When generalizing our findings to lower-volume institutions or different perioperative care settings, caution is warranted. All procedures in this study were performed by experienced endocrine surgeons in a high-volume tertiary referral center using standardized surgical techniques, which may have contributed to the relatively low incidence of permanent hypoparathyroidism observed in our cohort. External validation in larger, multicenter cohorts is needed before our proposed PTH threshold can be broadly applied across different surgical settings.

PTH measurement alone is insufficient for the management of postoperative hypocalcemia after thyroid surgery; therefore, combined assessment of serum PTH and calcium levels is generally recommended [8]. Several studies have shown that early postoperative serum calcium levels, most commonly measured on the first postoperative day, are associated with the risk of PH [7, 26, 27]. Reported thresholds range from ≤ 1.93 mmol/L (7.73 mg/dL) to ≤ 2.03 mmol/L (8.12 mg/dL), with sensitivities between 63.6% and 100% and specificities between 63% and 78.1% for predicting permanent hypoparathyroidism [26, 27].

In our cohort, postoperative first-day serum calcium levels were significantly lower in patients who developed permanent hypoparathyroidism compared with those who did not (8.1 vs. 8.7 mg/dL, *p* < 0.001). A calcium threshold ≤ 8.3 mg/dL predicted PH with a sensitivity of 72.2%, specificity of 74.7%, and an NPV of 98.6%. Although several studies have reported postoperative first-day serum calcium as an independent risk factor for permanent hypoparathyroidism [8, 26–28], in our analysis, lower calcium levels were significantly associated with permanent hypoparathyroidism in univariate analysis but did not retain independent significance in multivariate analysis.

Previous studies, including a meta-analysis, have reported higher rates of permanent hypoparathyroidism in patients undergoing thyroidectomy for large goiters and Graves’ disease [7]. In addition, age and sex have been suggested as potential contributors to parathyroid-related complications, particularly in the early postoperative period [7, 8, 25–29]. In the present study, however, patient- and disease-related factors—including age, sex, preoperative diagnosis, and thyroid size—were not significantly associated with the development of permanent hypoparathyroidism.

Thyroidectomy combined with lymph node dissection has been hypothesized to increase the risk of hypoparathyroidism due to inadvertent trauma or devascularization of the parathyroid glands. However, the impact of central lymph node dissection (CLND) on permanent hypoparathyroidism remains controversial. While several studies have reported no significant association between CLND and permanent hypoparathyroidism, others have suggested an increased risk [5, 22, 30]. In our cohort, no significant association was observed between CLND and the development of permanent hypoparathyroidism. Nevertheless, only 52 patients (10.4%) underwent CLND, which limits the statistical power to draw definitive conclusions regarding its contribution to this complication.

Hemithyroidectomy in the primary surgical setting leads to anatomical disruption due to tissue remodeling, fibrosis, and scar formation. However, the role of completion thyroidectomy in the development of PH is inconsistent. Several studies have advocated that thyroid surgery completion is a risk factor for PH, but this association has not been duplicated in other studies [31–33]. We found on multivariate analysis that completion thyroidectomy is an independent risk factor for developing PH.

The presence of parathyroid tissue in the surgical specimen is considered a marker of inadvertent parathyroidectomy and has been reported in 6.4% to 31.1% of thyroidectomy procedures [34, 35]. In our cohort, parathyroid glands were identified in 19.3% of thyroidectomy specimens, which is consistent with previously published data. Notably, the presence of parathyroid tissue in pathological specimens was significantly more frequent among patients who developed permanent hypoparathyroidism compared with those who did not (55.6% vs. 17.9%, *p* < 0.001). Although this association did not retain statistical significance in multivariate analysis (odds ratio: 3.44, 95% confidence interval: 0.97–12.5, *p* = 0.056), the observed trend supports previous reports suggesting an association between inadvertent parathyroidectomy and the development of permanent hypoparathyroidism [5, 31].

Previous studies have suggested that surgical experience is associated with a reduced incidence of postoperative hypoparathyroidism [36, 37]. Although surgical volume was not explicitly stated in this study, all surgeons performed more than 50 thyroidectomies annually. The relatively low incidence of PH observed in our study may be related to surgical experience.

Two principal strategies for postoperative calcium management have been described: parathyroid splinting and parathyroid stimulation [29]. The parathyroid splinting approach is based on the concept that calcium and vitamin D supplementation reduce parathyroid metabolic demand by maintaining the glands in a resting state, thereby decreasing symptomatic hypocalcemia and postoperative hospital stay. However, its effect on long-term parathyroid recovery remains controversial, as some studies suggest that supplementation facilitates recovery, whereas others report potential overtreatment and delayed parathyroid function with routine calcium and vitamin D administration [38–40].

In contrast, the parathyroid stimulation approach aims to promote parathyroid recovery through mild hypocalcemia, thereby stimulating calcium-sensing receptors, while maintaining serum calcium levels within slightly subnormal ranges to minimize hypocalcemic symptoms. Despite these theoretical advantages, neither strategy has been conclusively validated, and current guideline recommendations regarding postoperative calcium and vitamin D supplementation are largely based on low-quality evidence [13, 41].

In our institution, postoperative calcium and vitamin D supplementation is guided by an individualized, patient-specific approach rather than a rigid standardized protocol. Decisions regarding supplementation are primarily based on the presence of hypocalcemic symptoms, as well as postoperative serum calcium and parathyroid hormone levels.

### Limitations

This study has several limitations. First, owing to its retrospective design, the potential for both selection and information bias cannot be fully excluded, despite careful data collection, the use of standardized electronic medical records, and well-defined inclusion and exclusion criteria. Second, given the relatively low number of permanent hypoparathyroidism events (*n* = 18), the multivariable model may be susceptible to overfitting and parameter instability, and the estimated odds ratios with their wide confidence intervals should be interpreted with caution. External validation in larger, prospective cohorts is warranted before the proposed thresholds and multivariable associations are applied in clinical practice.

Detailed data regarding parathyroid autotransplantation and quantitative assessment of parathyroid preservation, such as the Parathyroid Gland Remaining In Situ (PGRIS) score, were not consistently available and therefore could not be reliably analyzed [42].

As this study was conducted at a single high-volume tertiary referral center, the findings may not be fully generalizable to lower-volume institutions or to community hospitals. In addition, this study focused exclusively on permanent hypoparathyroidism, which represents a clinically more relevant long-term complication after thyroid surgery. Data on transient hypoparathyroidism were not analyzed because early postoperative calcium and parathyroid hormone measurements were not consistently available for all patients owing to the retrospective design. Including transient hypoparathyroidism under these circumstances could have introduced a misclassification bias.

Postoperative calcium and vitamin D supplementation followed an individualized, patient-specific approach rather than a standardized protocol, which limited our ability to assess the independent impact of supplementation on biochemical outcomes. Finally, potential confounding factors, such as preoperative vitamin D status and baseline serum calcium levels, were not evaluated, which may have influenced postoperative biochemical outcomes and the interpretation of permanent hypoparathyroidism.

## Conclusion

Permanent hypoparathyroidism (PH) is a clinically significant complication of thyroid surgery, with important implications for long-term patient management. In this retrospective cohort study, we observed a PH incidence of 3.6%, consistent with findings from other high-volume endocrine surgery centers. A postoperative day 1 PTH level of ≤ 7 pg/mL was independently associated with PH and demonstrated an exceptionally high negative predictive value (99.4%), supporting its clinical utility as a reliable tool for ruling out PH rather than for confidently predicting it, given its modest positive predictive value (27.7%). Although low serum calcium levels (≤ 8.3 mg/dL) and the presence of parathyroid tissue in the surgical specimen were significantly associated with PH in the univariate analysis, they did not retain significance in the multivariate model. Completion thyroidectomy was also independently associated with PH. Given the limited number of events (*n* = 18), these multivariable findings warrant validation in larger, prospective cohorts. Overall, these findings underscore the value of early postoperative biochemical monitoring and meticulous surgical technique in minimizing the risk of permanent hypoparathyroidism.

## Data Availability

The datasets generated during and/or analysed during the current study are available from the corresponding author on reasonable request.

## Declarations

Part of the data included in this manuscript was presented as an oral presentation at the 12th National Congress of Endocrine Surgery, held in Antalya, Turkey, between February 13 and 16, 2025.
